# Evolving roles of clerkship directors: have expectations changed?

**DOI:** 10.1080/10872981.2020.1714201

**Published:** 2020-01-11

**Authors:** Gary L. Beck Dallaghan, Cynthia H. Ledford, Douglas Ander, John Spollen, Sherilyn Smith, Scott Graziano, Susan M. Cox

**Affiliations:** a Office of Medical Education, University of North Carolina School of Medicine, Chapel Hill, NC, USA; b Department of Internal Medicine, Oakland University William Beaumont School of Medicine, Rochester, MI, USA; c Department of Emergency Medicine, Emory University School of Medicine, Atlanta, GA, USA; d Department of Psychiatry, University of Arkansas for Medical Sciences, Little Rock, AR, USA; e Department of Pediatrics, University of Washington School of Medicine, Seattle, WA, USA; f Department of Obstetrics and Gynecology, Loyola University Chicago Stritch School of Medicine, Chicago, IL, USA; g Department of Medical Education, Dell Medical School-The University of Texas at Austin, Austin, TX, USA

**Keywords:** Medical students, medical education, accreditation, clinical clerkship, faculty

## Abstract

Background: Physician educators directing medical student programs face increasingly more complex challenges to ensure students receive appropriate preparation to care for patients. The Alliance for Clinical Education (ACE) defined expectations of and for clerkship directors in 2003. Since then, much has changed in medical education and health care. Methods: ACE conducted a panel discussion at the 2016 Association of American Medical Colleges Learn Serve Lead conference, soliciting input on these expectations and the changing roles of clerkship directors. Using workshops as a cross-sectional study design, participants reacted to roles and responsibilities of clerkship directors identified in the literature using an audience response system and completing worksheets. Results: The participants represented different disciplines of medicine and ranged from clerkship directors to deans of curriculum. Essential clerkship director qualifications identified by participants included: enthusiasm, experience teaching, and clinical expertise. Essential tasks included grading and assessment and attention to accreditation standards. Participants felt clerkship directors need adequate resources, including budget oversight, full-time clerkship support, and dedicated time to be the clerkship director. To whom clerkship directors report was mixed. Clerkship directors look to their chair for career advice, and they also report to the dean to ensure educational standards are being met. Expectations to meet accreditation standards and provide exemplary educational experiences can be difficult to achieve if clerkship directors’ time and resources are limited. Conclusions: Participant responses indicated the need for a strong partnership between department chairs and the dean’s office so that clerkship directors can fulfill their responsibilities. Our results indicate a need to ensure clerkship directors have the time and resources necessary to manage clinical medical student education in an increasingly complex health care environment. Further studies need to be conducted to obtain more precise data on the true amount of time they are given to do that role.

## Introduction

Educating students during the clinical phase of undergraduate medical education has become increasingly complex due to various medical school curricular changes, including the shortening of many discipline-specific clinical placements (henceforth referred to as clerkships), new areas of curricular focus, changes in health care delivery systems, and numerous additional or revised accreditation requirements [1,2]. While clinician educators have become increasingly recognized and valued as contributors to the success of an academic medical center [3–6], the expectations and roles of clinician educators who have significant administrative responsibilities are sometimes ambiguous.

The Alliance for Clinical Education (ACE) is an organization comprised of representatives from eight clerkship organizations representing emergency medicine, family medicine, internal medicine, neurology, obstetrics and gynecology, pediatrics, psychiatry, and surgery. The representatives typically have leadership roles within their discipline-specific organization. In 2003, ACE provided a collaborative statement endorsed by its member organizations on the expectations of, and for, clerkship directors [7]. The qualifications included knowledge of the education milieu, general leadership skills, teaching skills, clinical skills, career advising skills, and educational leadership skills.

Since that time additional publications have followed that further clarify resources and roles [6], needs [8], as well as special subgroups of clerkship directors, such as emergency medicine [9] and the medicine sub-internship director [10]. In addition, the educational landscape has markedly evolved to include a new focus on competency-based education [11], longitudinal integrated clerkship models [12], utilization of new educational technologies [13–15], enhanced early and advanced clinical coursework [16], and a need to accommodate increasing class sizes [17], often requiring increased use of geographically separate clinical sites [18]. Liaison Committee on Medical Education (LCME) standards and elements have evolved since release of the 2003 ACE article [7], resulting in increased responsibilities and accountability for clerkship directors and their schools, including but not limited to monitoring duty hours, providing mid clerkship feedback, and clinical site equivalency.

Given the evolving landscape of undergraduate medical education and the myriad changes since 2003, updated expectations of clerkship director roles and responsibilities are needed. The objectives of this paper are to critically analyze the elements included in the previous collaborative statement in order to 1) identify and prioritize the job responsibilities of the clerkship director identified by Pangaro et al. [7], 2) outline the essential skills needed to succeed in those responsibilities, incorporating new models of learning, 3) identify the individual(s) to whom he/she might report and, therefore, from whom he/she would seek support, such as clarification of responsibilities and provision of, or negotiation for, resources and 4) address emerging trends and challenges facing clerkship directors.

## Methods

We used workshops as a research methodology for this work [19]. This methodological framework uses a cross-sectional study design. The session was designed with the specific intent of producing data to determine the evolving roles of clerkship directors. In line with workshops as research methodology, we chose the collaborative participant mode, where workshop presenters and participants worked together but the presenters guided discussions.

### Session development

Presenters were ACE representatives, each of whom are medical education leaders and many of whom hold assistant to executive dean roles at their medical school. All of the panelists had prior experience as a clerkship director. The panel was conducted at the 2016 Association of American Medical Colleges meeting. The meeting is attended by approximately 4,500 people who are involved in health care education, research, and patient care. Attendees can choose sessions they want to attend. Since there are so many overlapping breakout sessions, individuals come and go from sessions due to these schedule conflicts.

Using the position statements of ACE [7] and constituent organization publications [9,20], a panel presentation was developed to address the evolving role of the clerkship director. The panel presentations provided background information by first giving an overview of the required and recommended expectations of clerkship directors [6,7], emerging trends in medical education, essential and suggested qualifications of a clerkship director, necessary resources to support the clerkship, stakeholders, and measurable outcomes of clerkship director performance.

### Data collection

Questions were posed to all of the participants using Poll Everywhere software to capture responses submitted via electronic device. Anonymous responses collected included demographic data. Participants were asked to rank the 2003 clerkship director duties from the ACE position statement based on current norms [7]. Table 1 includes the Poll Everywhere questions posed throughout the presentation.

**Table 1.** Poll everywhere questions

1. Indicate which clerkship organization you most closely associate:
• Association for Surgical Education
• Association of Professors of Gynecology & Obstetrics
• Association of Directors of Medical Student Education in Psychiatry
• Clerkship Directors in Emergency Medicine
• Clerkship Directors in Internal Medicine
• Consortium of Neurology Clerkship Directors
• Council on Medical Student Education in Pediatrics
• Society of Teachers of Family Medicine
• None of the above
2. In which Group on Educational Affairs region are you from:
• Central
• Northeast
• Southeast
• Western
3. Is your medical school:
• Private
• Public
4. Please indicate your academic rank:
• Instructor/Lecturer
• Assistant Professor
• Associate Professor
• Professor
5. How many years have you been on faculty at your current institution?
6. Which of these have caused you the most headaches?
• Accreditation changes/LCME issues
• Increased class size, new sites, regional campuses
• Competition from DO/PA/APN/etc. programs
• Competency based curricular design
• Integrated curricular structures
• Educational technologies
• Something else
7. Which of the following describes the current status of resources needed to support for your clerkship?
• Missing support for many critical functions
• Missing support for some critical functions
• Sufficient support for all critical functions
• Meets most of the current functions
• Meets all current functions + support for new initiatives
8. Which of the following describes the current status of resources needed to support for your clerkship?
• Missing support for many critical functions
• Missing support for some critical functions
• Sufficient support for all critical functions
• Meets critical functions, as well as most other functions
• Meets critical and current functions, plus support for new initiatives
9. Who would best advocate on behalf of student needs?
• College
• Department
10. Who would best advocate for needs of faculty teachers?
• College
• Department
11. Who would best advocate for clerkship director needs?
• College
• Department
12. To whom MUST the clerkship director report? (rank order)
• Vice President of Health Sciences or Dean
• (Associate) Dean for Undergraduate Education
• Chair of Curriculum Committee at College
• Chairman of Clinical Department
• Vice Chair for Education within Clinical Department
• Other?
13. Who is the clerkship director’s best advocate? (single best)
• Vice President of Health Sciences or Dean
• (Associate) Dean for Undergraduate Education
• Chair of Curriculum Committee at College
• Chairman of Clinical Department
• Vice Chair for Education within Clinical Department
• Other?
14. What makes a Director excellent?
• Appropriate completion of their required clerkship tasks/duties?
• Involved in innovative changes across the curriculum?
• Consistently disseminates scholarly work?
• Recognized outside the institution as an educational leader?

During the session, participants were also asked to work in small groups at their tables. Worksheets were developed by the panel presenters. The worksheets were developed using criteria from the ACE position statement [7] as a guide, but also included space for participants to write in responses (See Appendix A for worksheet examples). Participants were asked to identify what roles and responsibilities of clerkship directors are still relevant and what additional duties have emerged since ACE’s statement [7] was published. Participants also identified what qualifications make a good clerkship director and the key stakeholders necessary to be successful as a clerkship director. Finally, metrics to measure the success of a clerkship director were identified.

The worksheets were completed in small groups at tables during the session. These small groups were comprised of as few as two people to as many as eight. We collected 22 worksheets at the close of the session. Data from all of the worksheets, whether completed or not, were included in the analysis.

Participants were informed that this information would be used to revise and refine a collaborative statement regarding the expectations of and for clerkship directors. The University of North Carolina Institutional Review Board deemed this exempt.

Data were analyzed using descriptive statistics for scaled items from the audience response system. Data from the worksheets were transcribed verbatim. The panel presenters participated in reviewing the collated data from the worksheets to identify themes. Through emails and conference calls, consensus was achieved by the team.

## Results

### Participant characteristics

The panel space was reserved for 150 participants. Because of the arrangement of the conference, individuals come and go from sessions due to overlapping breakout sessions. Therefore, obtaining an exact number of participants from this session was not feasible. The initial count midway through the panel was approximately 85 participants.

Sixty-two attendees completed at least one of the Poll Everywhere questions. Twenty-one worksheets were completed by small groups of at least two people during the session. Respondents were distributed geographically amongst the Association of American Medical Colleges (AAMC) Group on Educational Affairs (GEA) regions, with 12 from the Northeast, 14 from the Central, 7 from the southern and 9 from the Western, 20 did not respond. The number of respondents from private medical schools (n = 28) was similar to those from public schools (n = 26), and 8 did not respond. Of those responding, 19 were Professors, 14 were Associate Professors, and 10 were Assistant Professors while 13 reported no academic rank or did not respond. Thirty-four respondents reported no association with a specialty organization or did not respond, but the rest were distributed amongst the member organizations in ACE: pediatrics (n = 6), internal medicine (n = 6), family medicine (n = 5), obstetrics/gynecology (n = 4), surgery (n = 3), psychiatry (n = 2), neurology (n = 1) and emergency medicine (n = 1). The average number of years reported on faculty at their institution was 14.56 ± 10.

### Qualifications of clerkship directors

**Figure 1.**
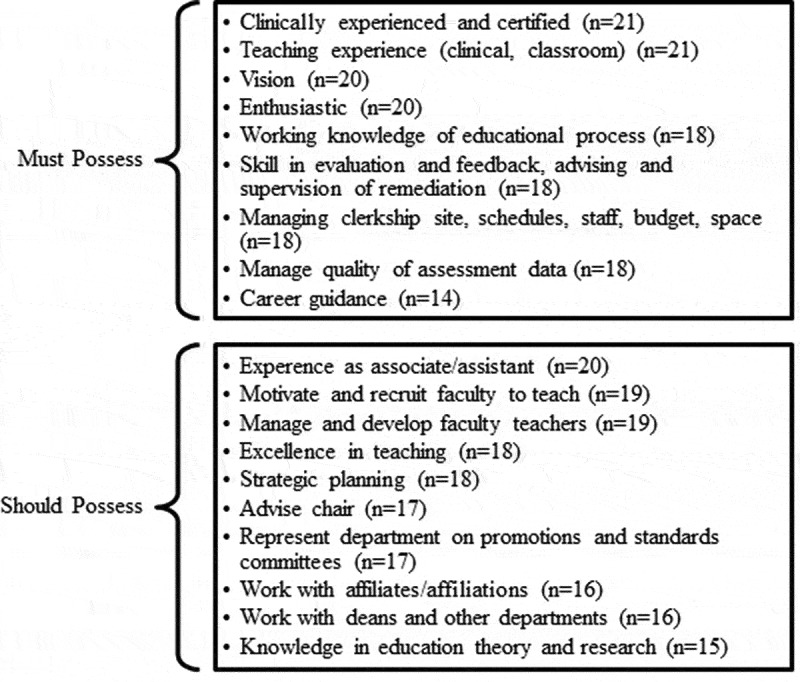
Participant expectations of clerkship director qualifications. Participant worksheets indicated which skills a clerkship director must possess and skills that they should possess. *n* indicates number of worksheets completed out of 22 total

In addition to what had been identified from the ACE position statement [7], small groups used the worksheets to identify additional qualifications of clerkship directors (Figure 1). The top-rated qualifications included teaching experience, clinical experience, enthusiasm, and being a visionary leader. Leadership skills were also essential, particularly for managing the clerkship operations, as well as the quality of assessment data.

**Figure 2.**
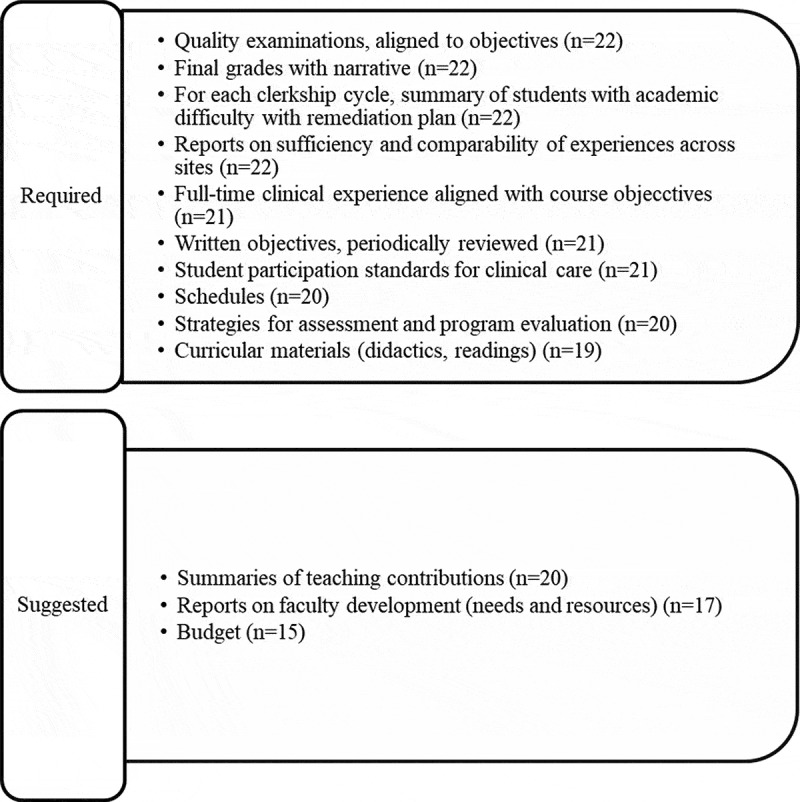
Essential tasks/products of the clerkship director. Participant worksheets indicated which tasks or products are a required or suggested responsibility of the clerkship director. *n* indicates number of worksheets completed out of 22 total

A Poll Everywhere question asked what makes an excellent clerkship director; most frequent responses were appropriately completes required clerkship tasks (n = 18), disseminating scholarly work (n = 17), and innovating changes across the curriculum (n = 17). Data obtained from the worksheets further detailed the required and suggested tasks of a clerkship director. Figure 2 summarizes these items. Of those previously identified by ACE [7], assistance with residency applications was rated as required by 11 groups, 7 as suggested, and 4 that it should not be an expectation at all. Participants were also split on to whom the clerkship director should report (9 required, 11 suggested) and making recommendations for changes in clerkship design or methods (13 required, 9 suggested).

### Resources

Participants were asked about the current state of resources needed to support their clerkship. A list was identified from Pangaro et al [7]. Based on the audience responses, they felt they were missing support for critical functions (n = 25) or they lacked support for many critical functions (n = 9). Only 12 respondents reported they had sufficient resources for all critical functions, three reported resources met the needs for most of the current functions and one reported that resources were available for all current functions as well as support for new initiatives. Twelve did not respond to this question.

The small groups rated which resources were necessary to administer the clerkship (Table 2). Of note, enough time to complete duties, as well as extra time to visit training sites, were identified as critical resources by all participants. Although initially listed as a suggested resource in the presentation, specific time allocation to be clerkship director was rated a must by 15 of the small groups with a recommendation the amount of time allocated for the role should be scaled based on the size of the clerkship.

**Table 2.** Resources needed for clerkship directors

Resource lists	Must	Should	Delete	Uncertain
Control over resources and budget	21			
Administrative support for clerkship management (1.0 FTE)	21			
Space for self, staff and teaching	21			
Access to new technologies and consultants	21			
Protected time at least .25 FTE	21			
Time and resources to visit other sites	21			
Assistant (coordinator) as ‘first contact’	20			1
Material resources (phones, computers, copies, etc.)	20	1		
Additional time and support for each course	20			1
Secretarial support for patient care issues	19	2	1	4
Time for research and development	4	17		
Access to statistical and informatics consultants	4	17		
Departmental committee (to review goals, strategies and students)	13	16		2
Time for committee work and personal development	6	13	1	
Time for evaluation and feedback to teachers	9	11		

Numbers reflect the number of worksheets identifying these resources.

### Stakeholders

When asked to respond to the question ‘who is the clerkship director’s best advocate’, 40 Poll Everywhere respondents chose the assistant/associate dean for undergraduate medical education, four chose the chair of the curriculum committee while only one chose the Chair of the Department. Responses about who the best advocate for clerkship directors is, the college of medicine or the clerkship director’s department, the college of medicine was favored for the clerkship director’s needs (39 for the college, nine for the department) and for student needs (34 for the college, seven for the department) but not for the needs of faculty teachers (26 colleges, 21 departments).

Small groups listed multiple stakeholders relying on the clerkship directors. As part of this exercise, the small groups were asked to indicate if the clerkship director should report directly to the stakeholder they identified. The groups indicated the clerkship director should report to the dean or medical education dean (n = 9) and the chair (n = 8). The curriculum committee was listed as a stakeholder by several groups, but only one indicated the clerkship director should directly report to that body.

## Discussion

The role of clerkship director continues to evolve in the face of an increasingly complex administrative milieu. Using a workshop as the research methodology, we identified and prioritized the current job responsibilities of the clerkship director with workshop participants. The essential skills needed to succeed in those responsibilities were outlined, incorporating new models of learning.

We also identified the individual(s) to whom clerkship directors might report and, therefore, from whom he/she would seek support, such as clarification of responsibilities and provision of, or negotiation for, resources. New findings addressed emerging trends and challenges facing clerkship directors such as a decreased priority on career advising and the increased importance of innovation and scholarship.

The 2003 statement from ACE suggested that the selection of clerkship director be regarded as an ‘implied contract between the clerkship director and the department chair’ [7]. Given the evolving educational landscape and the increase in cross-departmental, site, and organizational structures, there is uncertainty regarding to whom the clerkship director reports and prioritization of job responsibilities and roles. The small group comments indicated that the clerkship director should report to both the department chair and the dean’s office. This was a significant change from the past when the department chairs were the clear authority. The department chair was identified as able to ensure the clerkship director had protected time to fulfill teaching and administrative requirements.

As a new finding, the dean’s office was identified as important to the guidance needed to ensure the quality of the medical student experiences and to meet accreditation requirements. Therefore, chairs and the dean’s office need to have a much more deliberate and collaborative relationship related to undergraduate clinical medical education to ensure clerkship directors are given both the time and resources necessary to succeed.

The participants’ perception was that clerkship directors do not have sufficient support to accomplish all of the critical tasks of their work. This coupled with the self-defined view of excellence in this role as either completing all necessary tasks or completing all tasks while participating in innovation and scholarship may lead to faculty burn out and turn over [21,22]. Add to that conflicting perspectives of who clerkship directors should report places them in a challenging role, particularly since several of ACE’s member organizations report clerkship directors are junior faculty [23–26]. Although only a suggested skill by workshop participants, the authors believe budget negotiation skills may be a needed skill for clerkship directors to ensure adequate resourcing.

Increased medical school class sizes and accreditation requirements were commonly cited challenges facing clerkship directors. The number of students in medical schools in the US has increased by 7% over the past 4 years, with several new schools graduating their first classes [27]. In addition, training programs for other medical professionals such as physician assistants and advanced practice nurses have expanded to meet increased workforce demands [28]. With the increasing numbers of learners in clinical sites, patient care experiences can be impacted which may result in less optimal learning experiences for medical students, as evidenced by lower ratings for the clinical experience. This problem may also raise concerns about accreditation to ensure comparable experiences for medical students [2].

Meeting basic accreditation standards continues to require more and more of the clerkship director’s time. Clerkship directors must meet newer and more rigorous accreditation requirements such as ensuring and monitoring required clinical experiences, ensuring comparability of experiences across training sites, preparing faculty and residents to teach and evaluate students, and providing timely formative and summative feedback, as well as monitoring the learning environment, and managing student mistreatment issues [2].

Other accreditation standards directly apply to the clerkship director role, such as requirements to provide feedback to faculty (Element 4.4) and formative assessment and feedback to the student (Element 9.7). Obtaining meaningful assessments and feedback and ensuring that it is provided at the midpoint of a rotation has significant administrative and time implications for the clerkship director. Timely notification of final grades (Element 9.8) is a laudable goal, though multiple factors make this a challenge. Introduction of new skills and competencies, such as interprofessional collaborative skills (Element 7.9), to medical schools’ curricula impact clerkship directors who must now compress existing curricular content as well as effectively link new objectives to the wider medical education program (Element 8.2) and develop robust assessment of new skills (Elements 9.4 and 9.5). Other standards that apply to the entire medical education program, such as ensuring a learning environment that is conducive to education and professional behavior (Elements 3.5), also have a significant impact on the roles and responsibilities of clerkship directors.

While many of these standards may have been part of a medical education program the increased emphasis on the administrative aspects, assessment and introduction of new topics have added to the work of clerkship directors over the past 15 years. Of note, the number of standards that clerkship directors must ensure is met require time and resources. However, the LCME Standards [2] are devoid of recommendations for dedicated time for clerkship directors to meet these requirements. Unlike the Accreditation Council for Graduate Medical Education [29], the LCME has opted to not make specific recommendations for protected time for clerkship directors. Without that specification, chairs of departments are able to determine how much, if any, protected time clerkship directors receive to do this job.

In an era of electronic medical record systems, clerkship directors must ensure medical students have access to and know how to navigate and use the electronic health record across training sites. Additionally, direct observation of student performance is necessary if medical education is to be truly competency-based [30]. With the emerging promise of competency-based medical education, clerkship directors are challenged to provide multiple opportunities for direct observation of skills, coupled with high-quality assessment and feedback across different clinical contexts. This can be difficult to provide, given the growing number of learners, and is time-consuming for busy faculty who are under increasing demands for clinical productivity.

This report reflects findings from a panel conducted at the 2016 Association of American Medical Colleges Learn Serve Lead meeting. Participants attending the session had an interest in the topic and, therefore, likely have opinions based on shared experiences as clerkship directors, deans and other invested stakeholders. The results are limited in that attendees at this session may have had a vested interest in the topic, which could have biased results. We also were unable to capture specific details due to participants coming and going from the workshop. However, with the mix of representation from around the country and types of medical schools, the results of this session reflect a substantial evolution of the role, responsibilities, and expectations for and of the clerkship director.

## Conclusions

Expectations and challenges facing clerkship directors have only increased in the past 15 years since ACE published its guidelines for clerkship directors [7]. Changes to the education landscape, such as competency-based medical education, contribute to the evolving roles and responsibilities. Increasing expectations by department chairs and deans related to accreditation requirements, as well as the quality of educational experiences, can be difficult to meet if clerkship directors’ time and resources are limited. Participant responses indicated the need for a strong partnership between department chairs and the dean’s office to ensure clerkship directors have all the necessary resources to fulfill their responsibilities.

## Data Availability

Data are available by contacting the corresponding author at gary_beck_dallaghan@med.unc.edu.

## Attestation

Drs. Ander, Beck Dallaghan, Cox, Graziano, Ledford, Smith and Spollen have given final approval of this manuscript for publication.

## Inclusion Criteria

All of the authors developed the initial plan for this study and have participated in writing this manuscript.
